# Complete mitochondrial DNA genome of banded cichlid *Heros severus* Heckel, 1840 (Perciformes: Cichlidae)

**DOI:** 10.1080/23802359.2020.1787260

**Published:** 2020-07-09

**Authors:** Yan Chen, Chaojie Yang, Zhi Chen, Weixiang Tang, Zelin Lei, Yuxuan Du

**Affiliations:** College of Fisheries and Life Science, Hainan Tropical Ocean University, Sanya, PR China

**Keywords:** Cichlidae, mitochondrial genome, *Heros severus*

## Abstract

In this study, we sequenced the complete mitochondrial genome of *Heros severus* Heckel, 1840 (Perciformes, Cichlidae). This mitochondrial genome, consisting of 16,577 base pairs (bp), contains 13 protein-coding genes, 2 ribosomal RNAs, 22 transfer RNAs, and 2 noncoding control regions (control region and origin of light-strand replication) as those found in other vertebrates. Control region, of 917 bp in length, is located between tRNA^Pro^ and tRNA^Phe^. Within the control region, typical conserved domains, such as the termination-associated sequence (TAS), central, and conserved sequence blocks domains were identified. The overall base composition of the heavy strand shows 27.6% of T, 26.3% of C, 29.3% of A, and 16.8% of G, with a slight A + T rich feature (56.9%). The complete mitogenome data provide useful genetic markers for the studies on the molecular identification, population genetics, phylogenetic analysis, and conservation genetics.

The banded cichlid, *Heros severus* Heckel, 1840, is a common species distributed mainly in the South America (Lee et al. 1980). This freshwater fish as well as other congeneric species is distinguished by its unique shape and size and in particular their lips which are bulbous in appearance. Another characteristic is their demeanor which is sedentary and very similar to Discus fish expect when breeding. In brief, several studies have been carried out regarding the morphology of the aquarium species (Kullander 2003). However, only limited mitochondrial sequences have been published. Assessments of genetic information are essential to develop strategies for the conservation and rational utilization of *H. severus* resources. In this study, we presented the complete mitochondrial genome of *H. severus*, and some gene markers were further used for the phylogenetic status of this species.

The samples of *H. severus* were collected from the ornamental fish market in Sanya (18.11°N, 118.58°E) during August 2019. All five examined specimens have been deposited in the College of Fisheries and Life Science, Hainan Tropical Ocean University, Sanya, China (Voucher number: HTOU-CFLS-0807–HTOU-CFLS-0811). The HTOU-CFLS-0809 was used to extract total genomic DNA. The complete mitochondrial genome of *H. severus* has been deposited in GeneBank with accession number MT363636 (https://www.ncbi.nlm.nih.gov/nuccore/MT363636; 16,577 bp in length). It consists of 13 protein-coding genes, 22 *tRNA* genes, 2 *rRNA* genes, and 2 non-coding control region (control region and origin of light-strand replication). The arrangement of all genes is identical to that of most vertebrates (Wang et al. 2008; Chen 2013; Chiang et al. 2013). Most of the genes are encoded on the heavy strand (H-strand), except for the eight *tRNA* genes (-*Gln*, -*Ala*, -*Asn*, -*Cys*,-*Tyr*, -*Ser*, -*Glu*, and -*Pro*) and one protein-coding gene (ND6). The overall base composition is 27.6% for T, 26.3% for C, 29.3% for A, and 16.8% for G, with a slight A + T-rich feature (56.9%). Except for COI, ND4, and ND6 starting with GTG, the remaining 10 protein-coding genes start with ATG. It is important to note that some of the protein-coding genes (five of 13 genes) are inferred to terminate with an incomplete stop codon (ND2, COII, COIII, ND3, and ND4), with five (ATPase8, ATPase6, ND4L, ND5, and Cyt *b*) sharing TAA and three (ND1, COI, and ND6) using TAG as a stop codon, respectively. These features are common among vertebrate mitochondrial genome, and TAA is supposed to be appeared via posttranscriptional polyadenylation (Ojala et al. 1981). The longest one is *ND5* gene (1839 bp) among protein-coding genes, whereas the shortest is ATPase 8 gene (168 bp). The non-coding control region (D-loop) is 917 bp in length, and is located between tRNA^Pro^ and tRNA^Phe^. Within D-loop, a termination-associated sequence (TAS), conserved sequence blocks (CSB 1 and CSB 2), and several areas of highly conserved sequence (C, D, and F Box) were detected. The 2 ribosomal *RNA* genes, 12S rRNA (948 bp), and 16S rRNA (1677 bp) are located between tRNA^Phe^ and tRNA^Leu^.

Phylogenetic relationships were constructed using NJ algorithm among 22 Cichlasomatinae (Cichlidae) species based on 12 H-strand mitochondrial protein-coding genes, 22 *tRNA*, and 2 *rRNA* genes (Figure 1). This phylogenetic tree shows that *H. severus* is more closely related to *Andinoacara pulcher* than other species.

**Figure 1.**
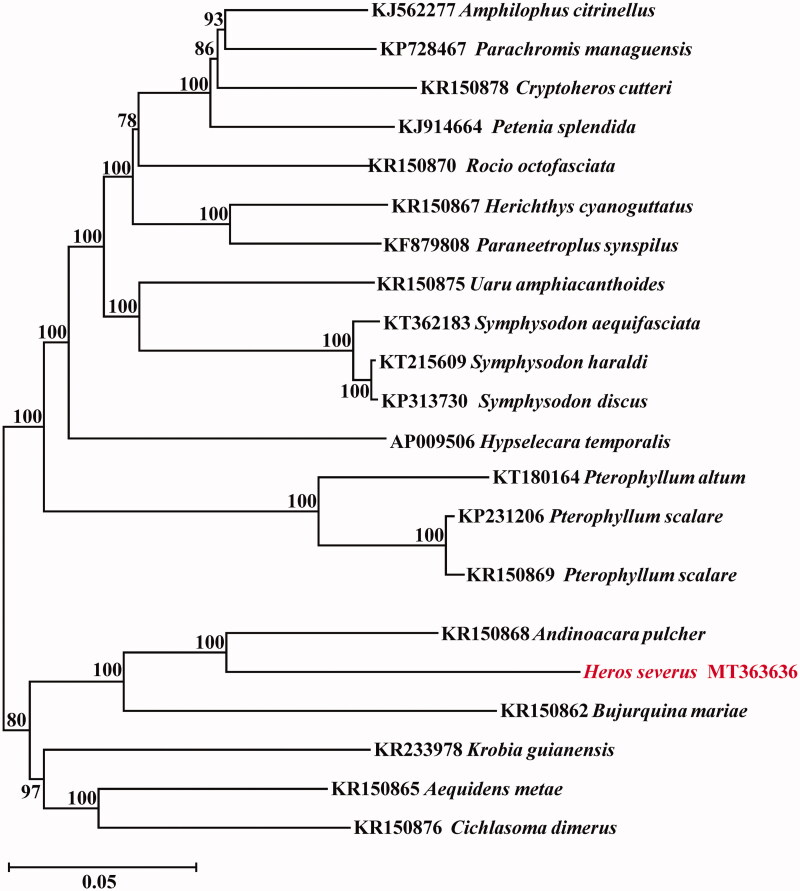
Phylogenetic relationships using NJ algorithm among same family species based on 12 H-strand mitochondrial protein-coding genes, 22 *tRNA*, and 2 *rRNA* genes.

## Data Availability

The data that support the findings of this study are openly available in GenBank of NCBI at https://www.ncbi.nlm.nih.gov, reference number MT363636.
